# How Do Apps Work? An Analysis of Physical Activity App Users’ Perceptions of Behavior Change Mechanisms

**DOI:** 10.2196/mhealth.7206

**Published:** 2017-08-03

**Authors:** Taylor H Hoj, Emarie L Covey, Allyn C Jones, Amanda C Haines, P Cougar Hall, Benjamin T Crookston, Joshua H West

**Affiliations:** ^1^ Computational Health Science Research Group Department of Health Science Brigham Young University Provo, UT United States

**Keywords:** mHealth, mobile apps, health behavior, smartphone

## Abstract

**Background:**

Physical activity apps are commonly used to increase levels of activity and health status. To date, the focus of research has been to determine the potential of apps to influence behavior, to ascertain the efficacy of a limited number of apps to change behavior, and to identify the characteristics of apps that users prefer.

**Objective:**

The purpose of this study was to identify the mechanisms by which the use of physical activity apps may influence the users’ physical activity behavior.

**Methods:**

This study used a cross-sectional survey of users of health-related physical activity apps during the past 6 months. An electronic survey was created in Qualtrics’ Web-based survey software and deployed on Amazon Mechanical Turk. Individuals who had used at least one physical activity app in the past 6 months were eligible to respond. The final sample comprised 207 adults living in the United States. 86.0% (178/207) of respondents were between the ages of 26 and 54 years, with 51.2% (106/207) of respondents being female. Behavior change theory informed the creation of 20 survey items relating to the mechanisms of behavior change. Respondents also reported about engagement with the apps, app likeability, and physical activity behavior.

**Results:**

Respondents reported that using a physical activity app in the past 6 months resulted in a change in their attitudes, beliefs, perceptions, and motivation. Engagement with the app (*P*<.001), frequency of app use (*P*=.03), and app price (*P*=.01) were related to the reported impact of the behavior change theory or mechanisms of change. The mechanisms of change were associated with self-reported physical activity behaviors (*P*<.001).

**Conclusions:**

The findings from this study provide an overview of the mechanisms by which apps may impact behavior. App developers may wish to incorporate these mechanisms in an effort to increase impact. Practitioners should consider the extent to which behavior change theory is integrated into a particular app when they consider making recommendations to others wishing to increase levels of physical activity.

## Introduction

Regular physical activity is effective in primary and secondary prevention of chronic diseases, including those relating to both physical and mental health outcomes [1]. It is associated with cardiovascular and metabolic health, reduced body mass, decreased risk of type 2 diabetes, optimal bone health, and strong motor control and physical functioning [2]. Obesity-related conditions, including heart disease, stroke, and type 2 diabetes, remain the leading causes of preventable death [3]. Consistently engaging in physical activity has also been linked to improved mental health status, as well as increased cognitive functioning and academic performance [4,5].

Despite clear and consistent guidelines establishing standards for physical activity, only half of the adults worldwide meet the recommended levels [6]. Inactivity is much higher in the United States where nearly 80% of adults fail to get the recommended amount of physical activity each week [7]. In light of the low global prevalence of physical activity and the strong connection to health outcomes, the current paradigm could rightly be considered a pandemic [8]. Promoting physical activity will require a sustained effort across multiple domains and disciplines, including behavioral science [8]. Efforts to date have included environment and policy changes [9], school and worksite interventions [10,11], and technology solutions [12-14]. Moving forward, our public health approaches may benefit from increased innovation and creativity [8], including an emphasis on mobile technologies with increased capacities for delivering timely and adapted promotion [15].

Smartphone apps have emerged as a potential tool for individuals seeking to increase levels of physical activity in an effort to improve health status [13-15]. Indeed, tens of thousands of health apps are available across all platforms and have now been downloaded billions of times [16]. The results from studies of these tools appear promising [17,18]. However, little is known about the behavior change techniques included in most physical activity apps [19]. It’s one thing to measure and observe that an app may increase levels of physical activity, but it’s another to understand the mechanisms to explain such effects [20]. These mechanisms may involve techniques inspired by behavior change theory, an accepted approach for increasing the effectiveness of physical activity interventions [8,21-23].

Despite the wide use of health-related physical activity apps by millions of Americans [24], only a few studies have considered the effectiveness of these apps at impacting the factors known to influence changes in behavior. Health behavior theory can inform behavior change interventions. The health belief model, for example, contains several primary constructs used to predict whether and why people take action to prevent, detect, or control disease outcomes. These constructs include perceived susceptibility, perceived severity, perceived benefits, and barriers to engaging in a behavior. Also addressed are cues to action, meaning internal or external factors that could trigger health behavior [25]. Social cognitive theory elucidates the interaction and impact of environment as it relates to influencing health behaviors. Cognitive influences on behavior (eg, knowledge, self-efficacy, and outcome expectancies) as well as the physical and social environment are core constructs [26]. The theory of planned behavior represents an attempt to predict behavior through impacting behavioral intentions. This is accomplished by changing attitudes, subjective norms, and self-efficacy [26].

The focus of several recent studies has been to analyze the content of physical activity apps and identify the extent to which behavior change theory is integrated [13,15,27-30]. Studies of this nature have provided an initial framework and context by which app potential can be evaluated. However, such studies are limited in that they only evaluate potential rather than actual impact, and there is no indication as to the role of these theory-based mechanisms of behavior change. For example, prominent theories such as the social cognitive theory and the health belief model emphasize the self-efficacy construct. Although attempts to label its inclusion in a physical activity app have been made recently, no empirical effort has been made to identify whether, in an app environment, it can be impacted and whether that can result in a change in physical activity-related behaviors. Constructs from the transtheoretical model [31-33] and the theory of planned behavior [34] also inform mechanisms whose impact should be measured, as each of these have been successfully used previously to influence physical activity. Provided the complexities relating to human behavior and in the context of physical activity, it may be that no one single theory adequately accounts for the most influential determinants. Therefore, a practical approach may involve a combination of distinct constructs and elements from each theory, effectively forming a polytheoretical approach [35]. The purpose of this study was to, first, identify whether and which behavior change mechanisms are impacted by using a physical activity app, and second, whether changes in these mechanisms also relate to physical activity behaviors.

## Methods

### Design

This study involved a cross-sectional survey designed to analyze the self-reported impact of using a physical activity app on the mechanisms of behavior change. This was done through a survey directed to individuals who had utilized at least one physical activity app within the last 6 months. The survey gathered information regarding mechanisms of behavior change informed by behavior change theory, app engagement and likeability, app usage, and self-reported physical activity behavior.

### Recruitment

The study sample comprised respondents who were recruited through Amazon Mechanical Turk (MTurk) and Turk Prime. MTurk is a crowdsourcing Internet marketplace that enables individuals and businesses to coordinate the completion of tasks, of which surveys are a common variety.

The sample was limited to respondents who were 18 years of age or older and residents of the United States. A total of 251 respondents completed the survey. The results for 10 respondents were excluded from the final sample because they reported not having used a health-related physical activity app in the past 6 months. Additionally, only respondents who completed all of the survey items were included in the final sample, which included a total of 207 respondents.

### Procedure

An electronic survey constructed through Qualtrics’ Web-based survey software was used to collect data through MTurk. The survey was available in MTurk for approximately 2 weeks with a US $1 incentive. The incentive was increased to US $2 after 2 weeks, and the survey was relaunched with Turk Prime to improve the response rate. As part of relaunching the survey using Turk Prime, an authenticator was built into the survey to prevent repeat respondents. Compensation was entirely based upon completing the survey and not on the quality of the responses.

### Measurement

Demographic information was gathered and respondents were asked to report their age, race, ethnicity, sex, highest level of education obtained, and annual household income (Table 1). Additional information relating to app usage was also gathered. This included the number of physical activity apps respondents had used in the past 6 months, how often each app was used, which apps they used most frequently, and the average price of the apps that they used. The average survey completion time was 5 min and 48 sec.

Respondents gave responses to a series of questions designed to measure the app’s impact on the mechanisms of behavior change. The questions were based on three prominent behavior change theories: social cognitive theory, the theory of planned behavior, and the health belief model. Items were developed to measure specific constructs within these theories. For example, “My belief that physical inactivity leads to disease” is a reflection of outcome expectancies, a fundamental construct of social cognitive theory. A full list of items and their corresponding theories are displayed in Table 2. These items were adapted from Cowan et al [13]. The adaptations were done to make them applicable for users’ perceptions of the apps’ impact. The items were then pilot-tested with 36 individuals. The pilot test comprised completing the survey in its entirety and then discussing each item with the pilot test takers to determine that each item was clearly written, understandable, and interpreted in the way it was intended.

A standard, 5-point Likert response scale was used to measure these items, ranging from Strongly disagree (−2) to Strongly agree (+2). A composite theory variable was constructed by summing the values of these 20 items, and the Cronbach alpha of this variable was .931. This variable provided a global assessment of the extent to which the apps impacted constructs believed to be important in influencing behavior change. A polytheoretical measure was determined to be in line with the viewpoint that multifactorial behaviors are too complex for any one single theory and may be best addressed with multiple theories [35]. The composite variable constructed in this study was not normally distributed, and a square root transformation was used to normalize it.

Five items related to the likeability and engagement (actual items displayed in Table 3) of the apps were also assessed using the same Likert scale. A composite variable was constructed to provide an assessment of the extent to which respondents liked and engaged with the apps. The Cronbach alpha for this scaled variable was .890. A square root transformation was used to normalize this scaled variable. Respondents were also asked to report about physical activity behaviors (actual items displayed in Table 4) that changed as a result of using the apps. These items were adapted from a recent study of physical activity apps [36]. The same Likert scale was used to measure physical activity. A composite variable was constructed with these 4 items, and the Cronbach alpha of this variable was .854. Again, a square root transformation was used to normalize this composite variable.

### Statistical Analysis

Stata version 14 was used to calculate all statistics. Descriptive statistics were calculated for each of the demographic, app usage, theory, engagement, and behavior variables. Multiple regression analysis was used to identify factors associated with behavior change theory constructs as well as with physical activity behavior, after controlling for potentially confounding variables.

## Results

### Demographics

The majority of respondents were between the ages of 26 and 54 years, with 45.9% (95/207) reporting their age between 26 and 34 years, and 40.1% (83/207) reporting their ages between 35 and 54 years (Table 1). Concerning race and ethnicity, 82.1% (170/207) of respondents reported being white and 94.2% (195/207) of respondents reported being of a non-Hispanic/Latino ethnicity. Females comprised 51.2 % (106/207) of respondents. Whereas the levels of education varied from less than a high school education to a professional degree, 63.8% (132/207) of respondents had either a 4-year degree or some college (not graduated) education. When asked about the number of physical activity apps used in the past 6 months, 60.9% (126/207) of respondents reported using only 1 physical activity app, whereas 29.0% (60/207) reported using 2 physical activity apps. Regarding frequency of physical activity app use in the past 6 months, 41.0% (85/207) of respondents reported using the apps daily, whereas 48.3% (100/207) of respondents reported using the apps multiple times a week. The most commonly used apps as reported by study respondents were Fitbit and MyFitnessPal, with 22.2% (46/207) of respondents reporting using Fitbit and 17.4 % (36/207) of respondents reporting using MyFitnessPal.

### Responses

A majority of respondents (58.0%, 120/207) reported “Strongly agree” that using the apps increased their desire to be healthy (Table 2). Similarly, 56.0% (116/207) strongly agreed that the apps increased their desire to be physically active. Respondents reported similar “Strongly agree” response rates for increased motivation, intention, goal setting desire, and ability to be physically active as a result of app use. A minority of respondents strongly agreed that the apps increased their belief that people important to them want them to be physically active (21.7%, 45/207), their knowledge of the diseases that are caused by physical inactivity (22.2%, 46/207), and their belief that physical inactivity leads to disease (24.6%, 51/207).

**Table 1:** Demographics (N=207).

Demographics			n (%)
**Age, in years**			
	18-25	17 (8.2)	
	26-34	95 (45.9)	
	35-54	83 (40.1)	
	55-64	11 (5.3)	
	65 or older	1 (0.5)	
**Race**			
	American Indian or Alaska Native	1 (0.5)	
	Asian	16 (7.7)	
	Black or African American	18 (8.7)	
	Native Hawaiian or Other Pacific Islander	2 (1.0)	
	White	170 (82.1)	
**Ethnicity**			
	Hispanic/Latino	12 (5.8)	
	Non-Hispanic/Latino	195 (94.2)	
**Gender**			
	Male	101 (48.8)	
	Female	106 (51.2)	
**Education level**			
	Less than high school	2 (1.0)	
	High school/General educational development	27 (13.0)	
	Some college (not graduated)	56 (27.1)	
	2-year college degree	28 (13.5)	
	4-year college degree	76 (36.7)	
	Master’s degree	15 (7.3)	
	Professional degree (JD, MD, etc)	3 (1.5)	
**Region of residence in the United States of America**			
	Northeast	36 (17.4)	
	Midwest	34 (16.4)	
	South	93 (44.9)	
	West	44 (21.7)	
**Annual household income^a^**			
	Less than 30,000	47 (22.7)	
	30,000-39,999	39 (18.8)	
	40,000-49,999	23 (11.1)	
	50,000-59,999	28 (13.5)	
	60,000-69,999	15 (7.3)	
	70,000-79,999	19 (9.2)	
	80,000-89,999	12 (5.8)	
	90,000-99,999	6 (2.9)	
	100,000 or more	18 (8.7)	

^a^All values are in 2016 US dollars.

**Table 2:** Responses to behavior change constructs (N=207). A composite behavior theory variable was computed by summing these variables, Cronbach alpha=.931.

Construct or mechanism of change^a^	n (%)				
	Strongly disagree	Somewhat disagree	Neither agree nor disagree	Somewhat agree	Strongly agree
My belief that physical inactivity leads to disease (outcome expectations)^b^	11 (5.3)	39 (18.8)	32 (15.5)	74 (35.8)	51 (24.6)
My belief that diseases related to physical inactivity are harmful (outcome expectancies)^b^	8 (3.9)	26 (12.6)	36 (17.4)	65 (31.4)	72 (34.8)
My belief that being physically active can prevent disease (behavioral belief)^c^	2 (1.0)	14 (6.8)	27 (13.0)	85 (41.1)	85 (41.1)
My belief that physical activity is important in preventing disease (behavioral belief)^c^	2 (1.0)	15 (7.3)	24 (11.6)	86 (41.6)	80 (38.7)
My ability to be physically active (self-efficacy)^b^	1 (0.5)	8 (3.9)	14 (6.8)	80 (38.7)	104 (50.2)
My confidence that I can be physically active (self-efficacy)^b^	1 (0.5)	5 (2.4)	8 (3.7)	104 (50.2)	89 (43.0)
My motivation to be physically active (behavioral attitudes)^c^	0 (0)	3 (1.5)	10 (4.8)	79 (4.8)	115 (55.6)
My desire to be physically active (behavioral attitudes)^c^	0 (0)	1 (0.5)	17 (8.2)	73 (35.3)	116 (56.0)
My intentions to be physically active (behavioral intention)^c^	0 (0)	1 (0.5)	13 (6.3)	81 (39.1)	112 (54.1)
My attitudes about the importance of physical activity in preventing disease (behavioral attitudes)^c^	1 (0.5)	13 (6.3)	25 (12.1)	87 (42.0)	81 (39.1)
My belief that people important to me want me to be physically active (subjective norm)^c^	9 (4.4)	31 (15.0)	50 (24.2)	72 (34.8)	45 (21.7)
My perception that many other people are physically active (situational perception)^b^	8 (3.9)	29 (14.0)	39 (18.8)	71 (34.3)	60 (29.0)
My knowledge of ways in which I can be physically active (knowledge)^b^	2 (1.0)	14 (6.8)	15 (7.3)	95 (45.9)	81 (39.1)
My knowledge of the diseases that are caused by physical inactivity (knowledge)^b^	15 (7.3)	43 (20.8)	36 (17.4)	67 (32.4)	46 (22.2)
My awareness of the benefits of being physically active (perceived benefits)^d^	1 (0.5)	8 (3.9)	22 (10.6)	88 (42.5)	88 (42.5)
My desire to be healthy (behavioral attitudes)^c^	0 (0)	1 (0.5)	12 (5.8)	74 (35.8)	120 (58.0)
The social support I have received for being physically active (reinforcement)^b^	7 (3.4)	35 (16.9)	45 (21.8)	67 (32.4)	53 (35.6)
The positive feedback I have received for being physically active (reinforcement)^b^	7 (3.4)	21 (10.1)	38 (18.4)	80 (38.7)	61 (29.5)
My desire to set goals to be physically active (attitude toward behavior)^b^	0 (0)	1 (0.5)	10 (4.8)	87 (42.0)	109 (52.7)
My ability to achieve my physical activity goals (self-efficacy)^b^	1 (0.5)	3 (1.5)	11 (5.3)	91 (44.0)	101 (48.8)

^a^All theory questions in the survey were preceded by this statement: “Now think about the physical activity/exercise apps that you have used in the past 6 months. Using the apps has increased”:

^b^Social cognitive theory.

^c^Theory of planned behavior.

^d^Health belief model.

**Table 3:** Responses to likeability and engagement items (N=207). A composite engagement variable was computed by summing these variables, Cronbach alpha=.890.

Item^a^	n (%)				
	Strongly disagree	Somewhat disagree	Neither agree nor disagree	Somewhat agree	Strongly agree
The app was useful.	0 (0)	1 (0.5)	4 (1.9)	67 (32.4)	135 (65.2)
The app was easy to use.	0 (0)	1 (0.5)	3 (1.5)	64 (30.9)	139 (67.2)
I enjoyed using the app.	0 (0)	2 (1.0)	13 (6.3)	68 (32.9)	124 (59.9)
I liked the app.	0 (0)	0 (0)	7 (3.4)	72 (34.8)	128 (61.8)
I would recommend the app to others.	0 (0)	1 (0.5)	5 (2.4)	72 (34.8)	129 (62.3)

^a^All engagement questions in the survey were preceded by this statement: “Considering the apps that you have used in the past 6 months”:

Regarding the app likeability and engagement (Table 3), most respondents (65.2%, 135/207, “Strongly agree” and 32.4%, 67/207, “Somewhat agree”) found the physical activity apps “useful.” Respondents reported similarly for “The app was easy to use,” “I enjoyed using the app,” “I liked the app,” and “I would recommend the app to others.”

More than half of respondents strongly agreed that apps influenced frequency (58.5%, 121/207) and consistency (58.9%, 122/207) of physical activity (Table 4). Whereas 46.4% (96/207) of respondents strongly agreed that the apps helped their actual goal setting to be physically active, 42.0% (87/207) strongly agreed that their intensity of physical activity increased as a result of using the apps.

### Regression Analysis

App engagement (*P*<.001), frequency of app use (*P*=.03) and app price (*P*=.01) were all positively associated with health behavior theory (Table 5). Health behavior theory and app engagement were positively associated (*P*<.001) with physical activity (Table 6).

**Table 4:** Responses to physical activity behavior items (N=207). A composite behavior change variable was computed by summing these variables, Cronbach alpha=.854.

Item^a^	n (%)				
	Strongly disagree	Somewhat disagree	Neither agree nor disagree	Somewhat agree	Strongly agree
My actual goal setting to be physically active	1 (0.5)	2 (1.0)	6 (2.9)	102 (49.3)	96 (46.4)
My frequency of physical activity	1 (0.5)	0 (0)	9 (4.4)	76 (36.7)	121 (58.5)
My intensity of physical activity	0 (0)	14 (6.8)	24 (11.6)	82 (39.6)	87 (42.0)
My consistency in being physically active	0 (0)	3 (1.5)	7 (3.4)	75 (36.2)	122 (58.9)

^a^All theory questions in the survey were preceded by this statement: “Now think about the physical activity/exercise apps that you have used in the past 6 months. Using the apps has increased”:

**Table 5:** Regression analysis and behavior change theory (N=207).

Variable	Coefficient (Standard error)	*t*	*P*
App engagement	.23 (0.04)	6.22	<.001
Frequency of app use	.39 (0.17)	2.27	.03
Price	.46 (0.18)	2.54	.01
Age	.05 (0.11)	0.46	.65
Gender	.22 (0.16)	1.31	.19
Education	.01 (0.06)	0.11	.91

**Table 6:** Regression analysis and physical activity (N=207).

Variable	Coefficient (Standard error)	*t*	*P*
Theory	.21 (0.028)	7.52	<.001
App engagement	.40 (0.074)	5.45	<.001
Frequency of app use	−.01 (0.067)	−0.02	.99
Price	−.01 (0.07)	−0.17	.86
Age	.01 (0.04)	0.27	.79
Gender	−.06 (0.06)	−0.98	.33
Education	.019 (0.023)	0.83	.41

## Discussion

### Principal Findings

The purpose of this study was to identify mechanisms of behavior change that are impacted by using a physical activity app. Second, we sought to explore the relationship between mechanisms of behavior change and self-reported actual changes in physical activity behaviors. The majority of respondents reported that apps had a favorable impact on their perceptions, attitudes, and beliefs. Physical activity apps certainly resulted in a marked increase in their desire to be healthy and motivation to be physically active. A recent review of app-based interventions reported that the method for increasing motivation to be physically active may include providing prompt and timely feedback to the user [37]. The positive impact on beliefs, attitudes, and perceptions may be useful for prioritizing features in future app-based interventions. These theory-based mechanisms have been shown in nontechnology settings to predict changes in behavior [38], which is corroborated by their impact in this study as well. By contrast, only a few respondents reported an increase in their knowledge of diseases related to physical inactivity as a result of app use. This could be the result of a lack of information available through the apps. This seems somewhat unlikely, provided that the previous research into health and fitness apps has shown that the provision of information is a dominant feature [13,15]. Alternative explanations may include that the respondents already had knowledge regarding the prevalence and effects of these diseases and that using the apps did little to improve upon their preexisting knowledge, or that the respondents were already motivated to be physically active and therefore had little interest in attending to any knowledge-related content. More importantly, it is not clear whether the lack of effect observed in this study is because of the apps truly being ineffective at impacting this aspect of behavior change theory, or whether the apps simply did not address it. A future study could be designed to study this aspect.

An association between the frequency of use and reported impact on the theory-based mechanisms of behavior change was observed. The exact reason for this relationship is not known. Some possible explanations may include the user-friendly nature of the apps that impacted theoretical constructs, increased user motivation, or higher user satisfaction. In a recent study of physical activity app users, respondents valued receiving push prompts and feedback [39], which may be indicators of engagement. Self-reported measures of app engagement and likeability were generally positive in this study, a finding that has also been reported broadly regarding health apps [37]. The relationship between mechanisms of change and self-reported app engagement is a novel finding. It is plausible that engaging apps influenced respondents’ perceptions of the overall impact of an app. It may also be the case that engaging with the apps allows for sufficient opportunities for impact. Whatever the reason, it may be of interest to practitioners and developers wishing to use apps to note the importance of engagement. Additionally, strategies to increase the frequency of use of the app should also be considered. Frequency of use continues to be a topic of interest for researchers in this space [40], but it stands to reason that an effective app will be more impactful if it is used more frequently.

Higher priced apps in this study were more likely to have a positive influence on the mechanisms of change, including constructs such as respondents’ attitudes, beliefs, and perceptions. A similar finding has also been reported in other studies of health and fitness apps, where apps with a higher cost were evaluated to have greater potential for influencing behavior change [15,41]. There could be several explanations for this correlation. It is possible that paid-for apps provide additional features or have higher quality programming and functionality allowing for greater behavior change theory integration. Additionally, respondents paying for apps may be more dedicated to using the apps because of their financial investment. Respondents who already regularly engage in physical activity may also consider a paid-for app a wise investment, as they will use it often. Similarly, the willingness of respondents to pay for physical activity apps may be representative of a stronger commitment or dedication to engage in physical activity. This finding may have implications for health professionals in that it might be advantageous for practitioners to recommend paid apps, as opposed to those that can be downloaded for free.

This study makes two unique contributions to extant literature. First is the identification of the theory-based mechanisms that are most impacted by using a physical activity app. Second is the connection between the theory-based mechanisms of change and physical activity behavior. The significant findings of the latter provide at least some validation of the major findings of this study. If there had been no association between the mechanisms of change and physical activity behavior, the study findings would be of little practical significance. In light of this finding, future efforts could focus on development-related questions to determine the most effective way to integrate and impact these theory-based mechanisms of change.

### Limitations

The limitations of this study should be considered when interpreting the findings. First, respondents in this study had limited diversity regarding race, ethnicity, education, and income. Respondents in this study were primarily white, educated, and had higher income status. This limitation is likely a reflection of the demographic using mTurk’s Web-based surveying system, which also tends to mirror these demographic characteristics [42]. Furthermore, a national survey of health app users revealed that most are young, educated, and have a higher income status [43]. Perhaps a future study could include a more diverse sample. Second, this study would be strengthened by collecting additional respondent data and information. For example, collecting information regarding respondents’ body mass index, health status, or motivations for using a physical activity app may have been useful to explore the extent to which such characteristics determine apps’ impact. Furthermore, additional validation of the survey is required before using it in new studies. Lastly, respondents’ use of apps and the attending impact was not measured longitudinally. Future studies may benefit from qualitative and quantitative designs that explore these relationships in a longitudinal fashion. Despite these limitations, this study represents an initial effort to understand the mechanisms by which physical activity may be impacted and strategies for both future app development and research design.

### Conclusions

The purpose of this study was to investigate the mechanisms by which changes in physical activity might occur when using a physical activity app. Findings indicate that increased engagement and use may be related to mechanisms of behavior change informed by behavior change theory. Furthermore, these mechanisms of change appear to be related to physical activity. Those wishing to develop physical activity apps may consider ways to integrate these mechanisms of change. Additionally, practitioners in search of apps for recommendation to improve physical activity behaviors should consider apps with an emphasis on these theory-informed mechanisms with more confidence, as they may be more likely to result in behavior change.

## Abbreviations

mTurk: Amazon Mechanical Turk
